# Molecular Docking and Fluorescence Characterization of Benzothieno[3,2-d]pyrimidin-4-one Sulphonamide Thio-Derivatives, a Novel Class of Selective Cyclooxygenase-2 Inhibitors

**DOI:** 10.3390/molecules19056106

**Published:** 2014-05-14

**Authors:** Mariarita Barone, Giovanna Pannuzzo, Andrea Santagati, Alfio Catalfo, Guido De Guidi, Venera Cardile

**Affiliations:** 1 Department of Pharmaceuticals Sciences, University of Catania, V.le A. Doria 6, Catania 95125, Italy; E-Mails: mariarita.barone@libero.it (M.B.); asantaga@unict.it (A.S.); 2 Department of Bio-medical Sciences, Section of Physiology, University of Catania, V.le A. Doria 6, Catania 95125, Italy; E-Mail: giopannuzzo@interfree.it; 3 Department of Chemical Science, Section of Photochemistry, University of Catania, V.le A. Doria 6, Catania 95125, Italy; E-Mails: acatalfo@unict.it (A.C.); gdeguidi@unict.it (G.D.G.)

**Keywords:** anti-inflammatory, antipyrine, cancer detection, COX-2, fluorescence

## Abstract

The aims of this study were: (i) to explore the structure-activity relationship of some new anti-inflammatory benzothieno[3,2-d]pyrimidin-4-one sulphonamide thio-derivatives **1**–**11**; and (ii) to evaluate the possibility of using the most active compounds as fluorescent probes to determine tumours or their progression. Therefore, to know the precise mechanism by which these compounds interact with cyclooxygenase (COX)-2 enzyme, a molecular docking study was carried out; to assess spectroscopic characteristics, their absorption and emission properties were determined. The results demonstrated that some derivatives of benzothieno[3,2-d] pyrimidine exhibit interesting anti-inflammatory properties related to interactions with active sites of COX-2 and are fluorescent. The antipyrine-bearing compound **4** displayed high COX-2 affinity (ΔG = −9.4) and good fluorescent properties (Φ_fl_ = 0.032). Thus, some members of this new class of anti-inflammatory may be promising for fluorescence imaging of cancer cells that express the COX-2 enzyme. Further *in vitro* and *in vivo* studies are needed to confirm this hypothesis.

## 1. Introduction

**Figure 1:**
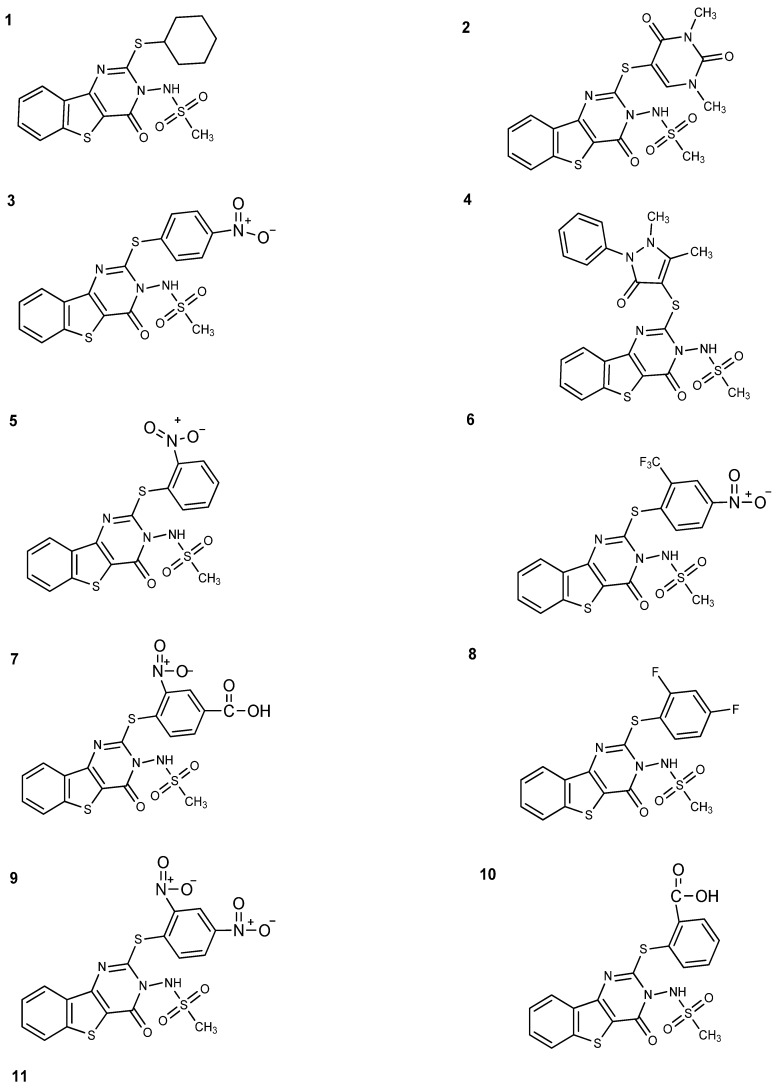
Benzo-thieno[3,2-d] pyrimidine derivatives **1**–**11**.

Over the last few years, we have focused our research in the synthesis of condensed heterocyclic derivatives, with the goal of developing efficient molecules with interesting pharmacological properties [1]. In a recent work, we described a new alternative synthetic method to produce new benzothieno[3,2-d]pyrimidin-4-one sulphonamide thio-derivatives **1**–**11** with anti-inflammatory properties (Figure 1), which is economically and environmentally very advantageous and characterized by the simplicity of the procedures, reduction of isolation and purification steps, time, costs, and waste production [2]. The anti-inflammatory activity of these derivatives was evaluated *in vitro* on a human keratinocyte NCTC 2544 cell line exposed to interferon (IFN)-γ and histamine, as well as on a monocyte-macrophage J774 cell line stimulated with bacterial lipopolysaccharide (LPS). These are two cell models particularly useful for reproducing the mechanisms involved in inflammation. To prove the biological efficacy of the new compounds, the expression of some mediators involved in the inflammation, such as cyclooxygenase (COX)-2, inducible nitric oxide synthase (iNOS), immuno-modulatory membrane molecules such as intercellular adhesion molecule-1 (ICAM-1), and the release of prostaglandins (PG)E_2_ and interleukin-8 (IL-8), were determined. The biological assays showed that, among the derivatives **1**–**11**, only the following compounds act as inhibitors of COX-2, iNOS and ICAM-1 expression (Table 1):

- *N*-[2-[(1,3-dimethyl-2,4-dioxo-1,2,3,4-tetrahydropyrimidin-5-yl)thio]-4-oxo[1]benzothieno[3,2-d]pyrimidin-3(4*H*)yl]methanesulfonamide (**1**);
- *N*-[2-[(1,5-dimethyl-3-oxo-2-phenyl-2,3-dihydro-1H-pyrazol-4-yl)thio]-4oxo[1]benzothieno [3,2-d]pyrimidin-3(4*H*)yl]methanesulfonamide (**2**);
- *N*-[2-[(2,4-nitrophenyl)thio]-4-oxo[1]benzothieno[3,2-d]pyrimidin-3(4*H*)yl]methanesulfonamide (**4**);
- *N*-[2-(cyclohexylthio)-4-oxo[1]benzothieno[3,2-d]pyrimidin-3(4*H*)yl]methanesulfonamide (**8**);
- *N*-[2-[(2,4-difluorophenyl)thio]-4-oxo[1]benzothieno[3,2-d]pyrimidin-(4*H*)yl]methane-sulfonamide (**9**);
- 2-({3-[(methylsulfonyl)amino]-4-oxo-3,4-dihydro[1]benzothieno[3,2-d]pyrimidin-2-yl}thio)benzoic acid (**10**).

At the same time, these compounds suppress the production of PGE_2_ and IL-8 in human keratinocytes NCTC 2544 and monocyte-macrophages J774 [2]. Therefore, the efficacy of benzo-thieno[3,2-d] pyrimidine derivatives in inhibiting COX-2 activity in inflamed cells indicates that this novel compound class may serve as potential therapeutic agents in inflammatory and proliferative disorders.

**Table 1:** iNOS and COX-2 IC_50_ values (μM) of human keratinocytes NCTC 2544 and mouse monocyte-macrophages J774 treated with interferon-γ plus histamine and lipopolysaccharides, respectively.

Compounds	NCTC2544		J774	
	iNOS	COX-2	iNOS	COX-2
**1**	7.0 ± 0.2	6.8 ± 0.5	7.3 ± 0.3	6.9 ± 0.9
**2**	7.0 ± 0.9	6.7 ± 0.3	6.5 ± 0.5	6.9 ± 0.5
**4**	5.0 ± 0.2	4.5 ± 0.5	4.8 ± 0.3	5.0 ± 0.1
**8**	5.8 ± 0.3	6.1 ± 1.5	6.5 ± 0.9	6.2 ± 0.6
**9**	8.0 ± 0.3	8.0 ± 1.0	7.8 ± 0.9	8.2 ± 0.4
**10**	6.5 ± 1.3	6.2 ± 0.8	6.5 ± 1.5	6.2 ± 0.5

Inflammation and carcinogenesis are interrelated phenomena [3]. The enzymes that mediate the constitutive synthesis of nitric oxide (NO) and PG from arginine and arachidonate, respectively, have relatively little influence on inflammation or on carcinogenesis [4]. In contrast, iNOS and COX-2 play critical roles in the response of tissues to injury or infectious agents [5]. These inducible enzymes are essential components of the inflammatory response, the ultimate repair of injury, and carcinogenesis [6,7]. Although physiological activity of iNOS and COX-2 may provide a definite benefit to the organism, aberrant or excessive expression of iNOS and/or COX-2 has been implicated in the pathogenesis of many disease processes, as septic shock, cardiomyopathy, acute and chronic neurodegenerative disease, rheumatoid arthritis, and carcinogenesis [8,9,10]. Clinical data suggest that over-expression of the COX-2 isozyme is associated with a diverse variety of human cancers that encompass gastric, breast, lung, colon, oesophageal, prostate, and hepatocellular carcinomas [11,12]. COX-2 enzymes are found at high levels in inflammatory lesions and tumours, whereas mainly absent in normal cells. The COX-2 signalling pathway is important in cancer because when it is activated, it can stimulate many key steps in cancer development, including cell division, inhibition of cell death, angiogenesis, and metastasis [13]. Recently, some authors reported that chronic inflammation drives the progression of colorectal cancer (CRC) and that COX-2 is one of the most important inflammatory genes involved in solid tumor metastasis. They demonstrated that store-operated Ca^2+^ entry is involved in the activation of transcription factors (CREB/NFAT) that are responsible for delivering EGF-mediated signals to evoke inflammatory cascades related to colorectal cancer tumorigenesis [14,15]. The alliance between COX-2 over-expression and cancer makes the COX-2 isozyme an attractive molecular target. Immense efforts were devoted to developing new molecules that are direct inhibitors of the enzymatic activity of COX-2. In a search for phytochemicals with anti-inflammatory activity, isodesacetyluvaricin, from the Formosan tropical fruit tree *Annona glabra*, exhibited potent anti-COX-2 activity, demonstrating to serve as a lead compound for targeting inflammatory diseases as well as angiogenesis and cancer metastasis [16]. The same authors demonstrated that 11-episinulariolide acetate from *Simularia flexibilis* shows functional inhibitory activity on cytoplasmic calcium concentration COX-2 and IL-8 gene expressions [17].

Several non-steroidal anti-inflammatory drugs (NSAIDs) were found to play an important role in cancer prevention providing evidence that COX-2 inhibitors may decrease the risk of developing cancer. Initially, COX-2-selective inhibitors were developed as anti-inflammatory agents with fewer gastrointestinal side effects compared to non-selective non-steroidal anti-inflammatory drugs (NSAIDs) [18]. These selective COX-2 inhibitors proved to be effective in inhibiting tumour growth in animal studies, and exhibited antiangiogenic activity *in vitro* that may contribute to their antineoplastic effects *in vivo* [19]. The antiangiogenic effects of COX-2-selective inhibitors and their ability to reduce haematogenous metastasis of COX-2-expressing tumours raised the possibility that they may be useful for treatment as well as for prevention of some cancers [19,20].

Recently, it was reported that the over-expression of COX-2 in cancer cells, relative to normal adjacent tissue where COX-2 is not expressed, constitutes a logical molecular diagnostic design strategy to discover non-invasive diagnostic agents to detect tumours, and the subsequent monitoring of disease progression and/or treatment efficacy [20,21]. A COX-2 inhibitory agent, fluorescent or coupled to a fluorescence tag, can serve as a potential COX-2 imaging emitting probe.

Since the derivatives **1**, **2**, **4**, **8**, **9** and **10** were shown to possess good anti-inflammatory activity, the aims of this study were to know the precise mechanism by which these new anti-inflammatory benzothieno[3,2-d]pyrimidin-4-one sulphonamide thio-derivatives interact with the COX-2 enzyme and to explore their structure-activity relationships through a molecular docking study. In addition, the possibility of using these derivatives to determine tumours or their progression was evaluated by spectroscopic characterization and determination of their absorption and emission properties.

## 2. Results and Discussion

### 2.1. Docking Study

In this study, the interaction of COX-2 active site residues with benzothieno[3,2-d] pyrimidine derivatives **1**–**11** (Figure 1) was described. Considering their good biological proprieties in the reduction of some anti-inflammatory parameters obtained on two cell models, an investigation of new derivatives/COX-2 interactions using structure-activity analysis was carried out. Molecular docking was performed on derivatives **1**–**11** of benzothieno[3,2-d] pyrimidine, that differed for the presence of a functional group; aryl or heterocyclic group. Nimesulide and naproxen were used as reference compounds.

We chose nimesulide (4-nitro-2-phenoxymethanesulphonanilide) as a prototype of selective COX-2 inhibitors. Nimesulide contains a polar nitro group that may hydrogen bond with Ser-530 and/or Tyr-385, in addition to a sulfone that may bind in the side pocket. Members of the methanesulfonanilide class of COX-2 inhibitors generally exhibit preferential COX-2 selectivity. These compounds are characterized as derivatives of alkylsulfonanilide. Nimesulide was the first member of this class to be discovered.

We chose naproxen [(*S*)-6-methoxy-α-methyl-2-naphthaleneacetic acid] for its high anti-inflammatory potency, confirmed by ΔG of our docking studies (−9.0 kcal/mol). Naproxen is a powerful non-selective non-steroidal anti-inflammatory drug that inhibits both COX-1 and COX-2 with comparable IC50 values [22]. Strong electron density was observed for a single orientation of naproxen binding within the COX-2 active site, making no contacts in the COX-2 side pocket or lobby region. As predicted by the mutagenesis data, the binding mode of naproxen is similar to that of other members of the 2-arylpropionic acid family of NSAIDs with the carboxylate group of naproxen participating in hydrogen-bonding interactions with Arg-120 and Tyr-355 at the base of the active site.

The structure of COX-2 consists of three distinct domains: an N-terminal epidermal growth factor (EGF) domain, followed by a membrane-binding motif and a C-terminal catalytic domain. This latter contains the cyclooxygenase and peroxidase active sites, with 87% identity and strict sequence conservation with respect to the cyclooxygenase active site. The structure of human COX-2 is expected to be very similar to the murine enzyme (mCOX-2) [23,24].

Our docking results indicated that the derivatives **1**, **2**, **4**, **8** and **10**, independently of their functional group, are able to bind the mCOX-2 active site while the compounds **3**, **5**, **6** and **7** have unfavourable interactions with mCOX-2.

The active site of mCOX-2 is a long, narrow hydrophobic channel extending from the membrane-binding region to the protein core. It is divided into three important region, the first is a hydrophobic pocket containing Tyr-385, Trp-387, Phe-518, Ala-201, Tyr-248 and Leu-352; the second region being entrance of the active site lined by the hydrophilic residues Arg-120, Glu-524, Tyr-355, and the third is a side pocket lined by His-90, Arg-513 and Val-523 [24].

In this study, ligand-protein docking methods were applied to predict the optimal positions, orientations and energies for interactions of the derivatives **1**–**11** with mCOX-2. The results suggested that the derivatives **1**, **2**, **4**, **8**, **9** and **10** have high binding energies (Table 2); unlike the derivatives **3**, **5**, **6** and **7** have low binding energies. It is possible to observe that the derivative **4** containing the antipyrine group showed the best binding energy: Δ*G* = −9.4 kcal/mol. Thus, there is a good correlation between the results of binding energies of the benzothieno[3,2-d] pyrimidine derivatives to mCOX-2 and the biological assays published in our previous article [2].

**Table 2:** Energy contributions for derivatives (**1**, **2**, **4**, **8**, **9**, **10**) with high binding energies.

Compound	Docking energies (ΔG) (kcal/mol)	Number of H-bonds	Donor/Acceptor Hydrogen bond	H-bond distance (Å)
Naproxen	−9.0	2	ARG-120:NH1	1.863
			TYR-355:OH 1	2.165
Nimesulide	−7.3	2	ARG-120:NH1	3.088
			TYR-355:OH 1	2.737
Derivative **1**	−7.9	2	ARG-120:NH1	3.149
			TYR-112:OH 1	2.806
Derivative **2**	−8.1	2	ARG-120:NH1	3.002
			TYR-112:OH 1	2.788
Derivative **4**	−9.4	3	ASN-382:NH2	2.867
			HIS-386:NH2	2.945
			THR-212:OH1	2.692
Derivative **8**	−8.9	1	ARG-120:NH1	2.882
Derivative **9**	−9.1	2	TYR-355:OH	2.875
			ARG-120:NH	2.876
Derivative **10**	−8.2	2	ARG-120:NH	2.970
			TYR-355:OH	3.120

**Figure 2:**
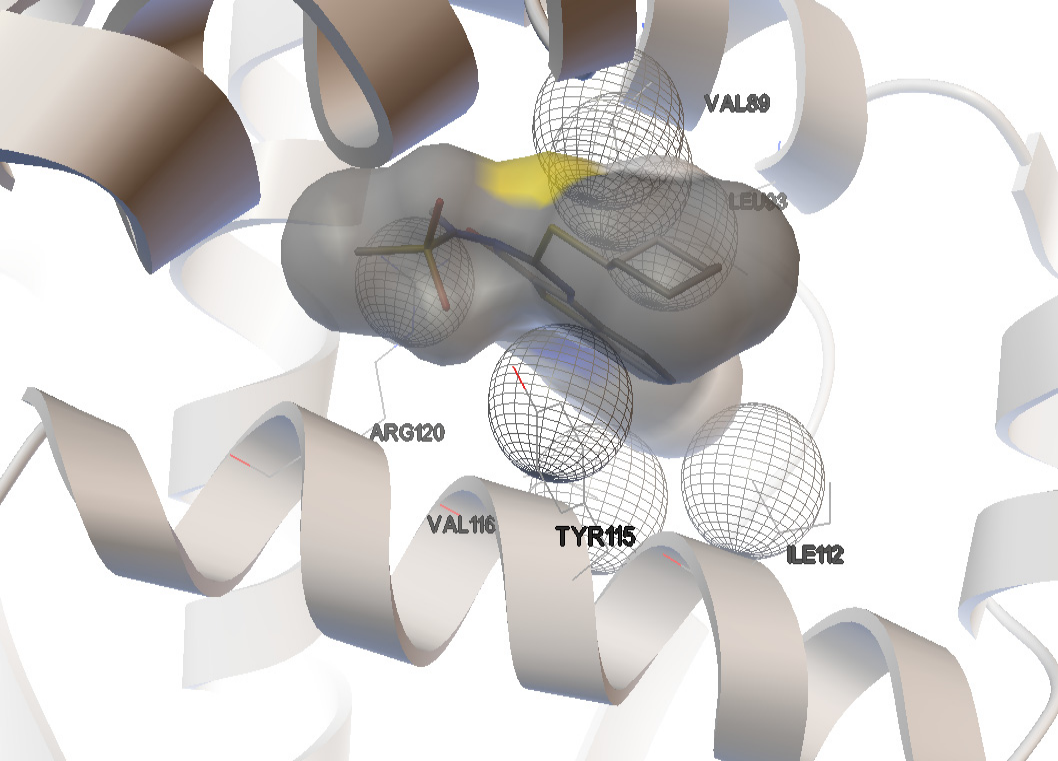
Binding of **1** into the active site of COX–2.

**Figure 3:**
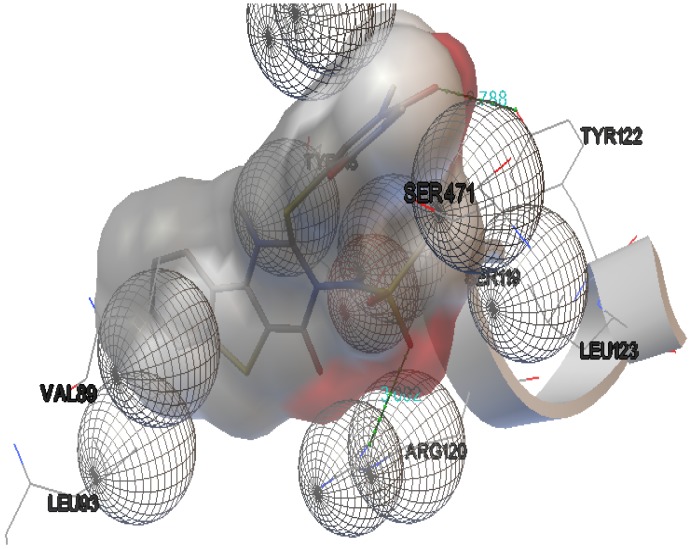
Binding of **2** into the active site of COX–2.

**Figure 4:**
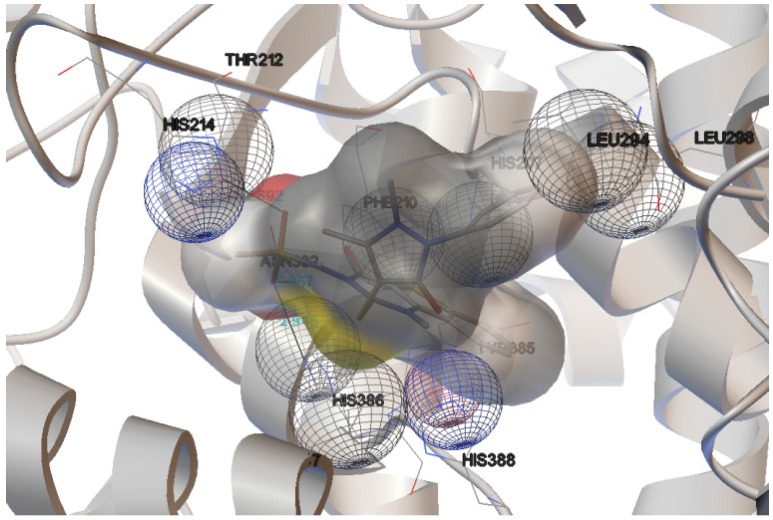
Binding of **4** into the active site of COX–2.

**Figure 5:**
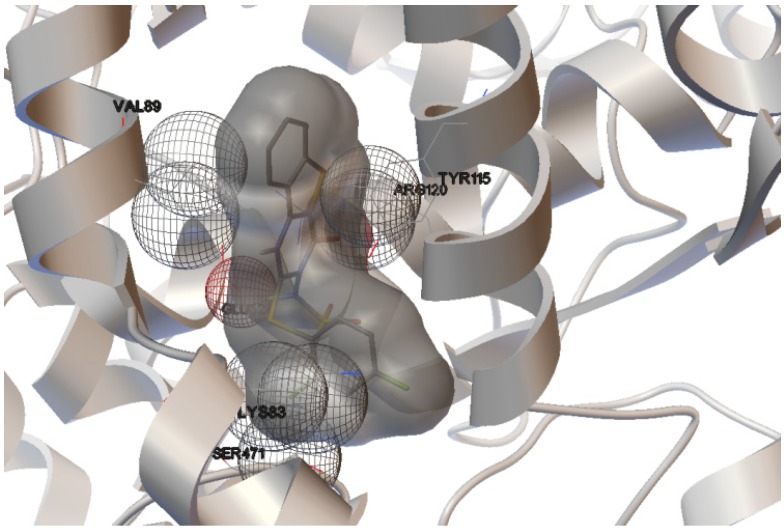
Binding of **8** into the active site of COX–2.

**Figure 6:**
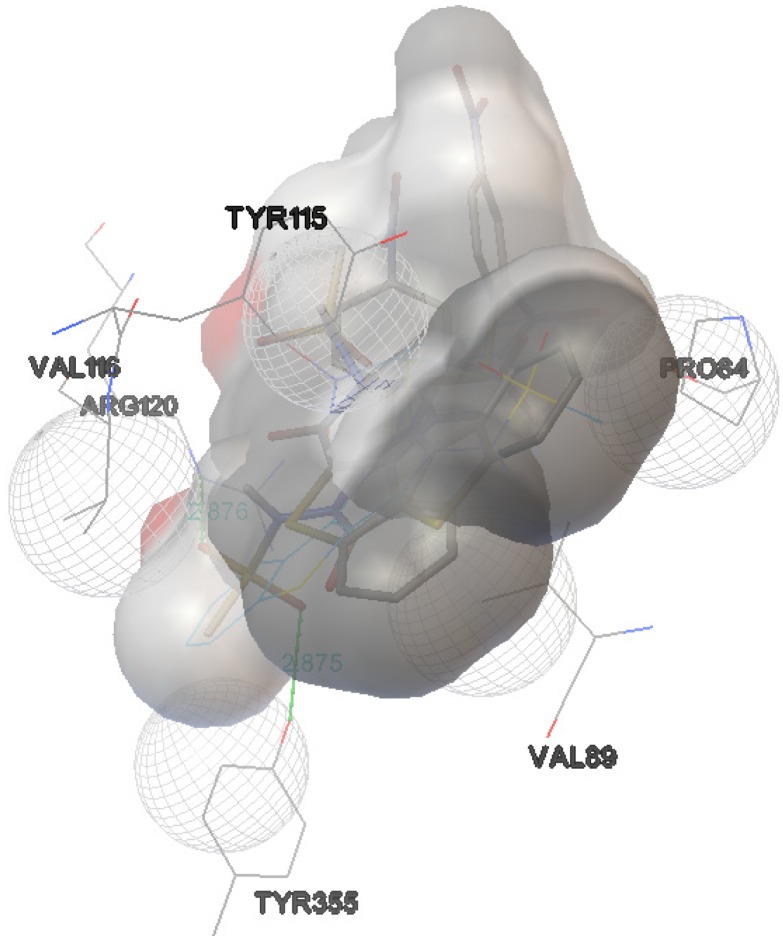
Binding of **9** into the active site of COX–2.

**Figure 7:**
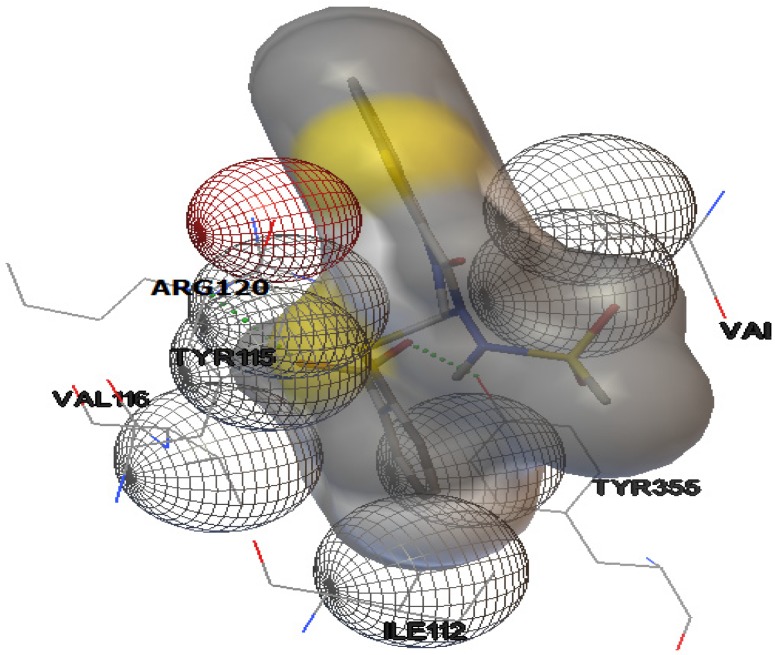
Binding of **10** into the active site of COX–2.

The interactions of compounds **1**, **2**, **4**, **8**, **9** and **10** with the mCOX-2 active site are shown in Figure 2, Figure 3, Figure 4, Figure 5, Figure 6 and Figure 7. Derivatives **1**, **2**, **6**, **8** and **10** exhibited interaction with the following enzyme amino acid residues: Lys-83, Pro-84, Val-89, Leu-93, Ile-112, Tyr-115, Val-116, Ser-119, Arg-120, Tyr-122, Tyr-355, and Ser-471. The compounds **1**, **2**, **6**, **8** and **10** were able to interact through a strong hydrogen bond with the amino acid residue Arg-120. Compounds **9** and **10** exhibit hydrogen bond with Tyr-355, whereas compound **1** and **2** with Tyr-112. In addition, the same compounds (**1**, **2**, **4**, **8**, **9**, and **10**) showed strong hydrophobic interactions of van der Waals with different amino acid residues in the mCOX-2 active site. By “strong” we mean hydrogen bonds that are able to control crystal and supramolecular structure effectively. This certainly includes O–H•••O=C, N–H•••O=C and O–H•••O–H. By “weak” we mean hydrogen bonds whose influence on crystal structure and packing is variable.

For derivative **1**, donor/acceptor hydrogen bonds: OH (Tyr 112)-SO_2_CH_3_ (**1**), NH (Arg 120)-CO (**1**).

For derivative **2**, donor/acceptor hydrogen bonds: OH (Tyr 112)-SO_2_CH_3_ (**2**), NH (Arg 120)-CO of the group 4-oxo-tetrahydropyrimidine (**2**).

For derivative **4**, donor/acceptor hydrogen bonds: NH (His 386)-SO_2_CH_3_ (**4**), NH (Asn 382)-SO_2_CH_3_ (**4**), OH (Thr 212)-SO_2_CH_3_ (**4**).

For derivative **8**, donor/acceptor hydrogen bonds: NH (Arg 120)-SO_2_CH_3_ (**8**).

For derivative **9**, donor/acceptor hydrogen bonds: OH (Tyr 355)-SO_2_CH_3_ (9), NH (Arg 120)-SO_2_CH_3_ (**9**).

For derivative **10**, the presence of carboxylate group changes the nature of donor/acceptor hydrogen bonds: OH (Tyr 355)-CO (**10**), NH (Arg 120)-CO (**10**).

Thus, the methanesulphonamide group present in all compounds was directed towards the channel where the active site is located. It forms a hydrogen bond with the hydroxyl group of Tyr-355 or Tyr-112 and amino of Arg-120. These results were comparable to interaction obtained by nimesulide and naproxen (Table 2). Kaur *et al.* demonstrated that, within the investigated group, 1,4 diaryl-substituted triazoles, 4-{2-[4-(4-chlorophenyl)-[1,2,3]triazol-1-yl]-ethyl}-benzenesulfonamide displayed highest COX-2 inhibitory potency and selectivity. Results of molecular docking studies revealed that COX-2 SO_2_NH_2_ pharmacophore in this compound is positioned in the secondary pocket of the COX-2 active site; with the nitrogen atom of the SO_2_NH_2_ group hydrogen bonded to Gln-192, and one of the oxygen atoms of SO_2_NH_2_ group forming a hydrogen bond to His-90 [25].

Moreover, the computational simulation studies showed that the compound **4** is the best candidate to inhibit COX-2, because its binding energy results the highest between all derivatives. The results demonstrated that compound **4** can interact with the following amino acid residues: His-207, Phe-210, Thr-212, His-214, Leu-294, Leu-298, Asn-382, Tyr-385, His-386, His-388 (Figure 4). The optimized complex of compound **4** and COX-2 showed three strong hydrogen bonds with the protons of His-386, Asn-382 and Thr-212.

The docking experiments performed on thiophene derivatives and some non-steroidal anti-inflammatory drugs (NSAIDs) indicated that His-386, His-388, His-214, His-207 amino acids are important residues that stabilize the conformations of compounds interacting with the binding pocket of COX-2 [26].

Finally, docking results revealed that the perpendicular orientation of thiophene and/or phenyl rings to the plane of imidazole rings of His residues (His-386, His-388, and His-207) was significant for locking the geometries of thiophene compounds in the COX-2 [26]. Our docking results confirmed that the amino acid residues His-386, His-388, His-214 and His-20 are very important for selective inhibition of COX-2. In conclusion, the data obtained by docking studies resulted perfectly in correlation with the results of biological assays previously published [2].

### 2.2. Spectroscopic Characterization

The absorption and emission properties of compounds are listed in Table 3. The excitation and emission spectra were determined by solution of **1**, **2**, **4**, **8**, **9**, and **10** in DMSO. Almost all compounds displayed peaks with absorption maxima at two different wavelengths (~340 and ~350 nm), and for all the compounds, the corresponding λ_em_ maxima were centred in the range 415–450 nm. In a study on yellow pigments, fomitellanols A (**1a**) and B (**2a**), and drimane-type sesquiterpenoid ethers of isocitric acid, cryptoporic acids P (**3**) and Q (**4)**, isolated from the fruiting bodies of *Fomitella fraxinea* (Polyporaceae) and analysed their biological activity against COX-1, COX-2, and 5-LO, the UV spectrum of 5-hydroxy-7-hydroxymethyl-1-isopropoxy-9b-methyl-9a,9b-dihydro-*H*-2-oxa-cyclopenta[d]acenaphthylen-9-one (**1a**) showed absorption bands at 224, 291, 347, and 385 nm, indicating the presence of a conjugated system [27].

**Table 3:** Absorption and emission properties of investigated compounds.

Derivative	ε (λ_max_)	ε (λ_max_)	Φ_fl_
**1**	10,991 (340)	9916 (354)	0.080
**2**	8304 (338)	7500 (352)	0.013
**4**	9784 (338)	8905 (352)	0.032
**8**	9662 (338)	8199 (350)	0.055
**9**	10,773 (338)	9855 (352)	0.007
**10**	9435 (334)	8643 (346)	0.087

ε, molar absorption coefficient, L/mol^−1^/cm^−1^; λ_max_, absorption maximum wavelength, nm; Φ_fl_, fluorescence quantum yield.

**Figure 8:**
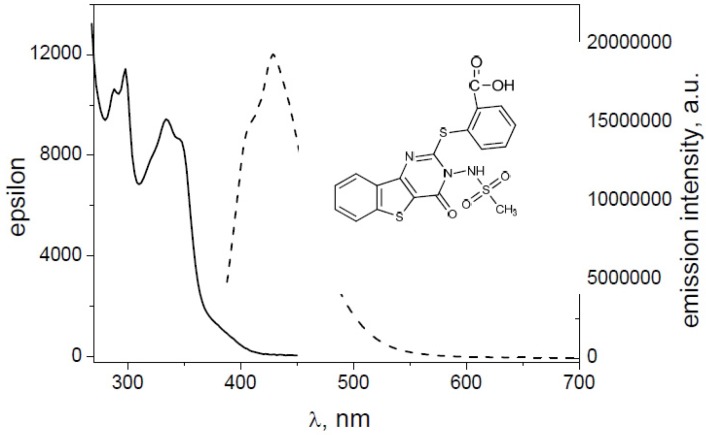
Absorbance (straight line) and emission (dashed line) spectra of **10**.

**Figure 9:**
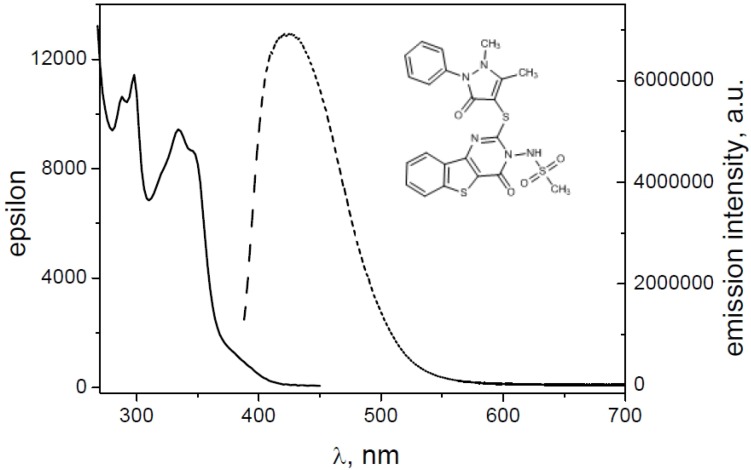
Absorbance (straight line) and emission (dashed line) of **4**.

**Figure 10:**
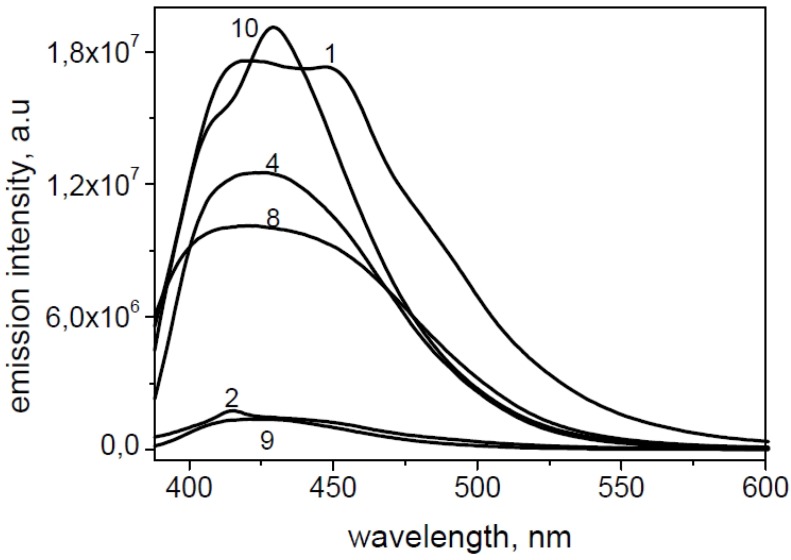
Emission spectra of **1**, **2**, **4**, **8**–**10**.

For a general example, in Figure 8 and Figure 9 the absorption and emission spectra of **10** and **4** are reported. In Figure 10 the emission spectra of the investigated compounds were shown. Excitation was fixed in the tail of the absorption spectrum to avoid re-absorption phenomena. All spectra were normalized for an absorbance in the exciting wavelength of 0.074. All the derivatives showed an important difference between λ_exc_ and λ_em_ (Stokes shift). Quantum yields (Φ) were determined to probe the environment affecting the sensitivity of the compounds (high quantum yield in nonpolar solvents or when bound to hydrophobic sites). Indeed, all tested compounds exhibited good fluorescence in organic solvent. Molar extinction coefficients (ε) were carried out for all compounds (in DMSO), resulting in similar values (about 10.000 L mol^−1^ cm^−1^); the highest value was displayed by the compound **1**, also if this latter was accompanied by a lower fluorescence intensity.

### 2.3. Fluorescence Microscopy

**Figure 11:**
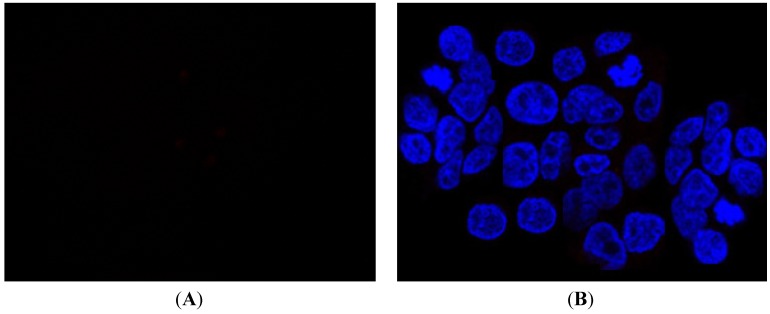
Fluorescent images of human epithelial colorectal adenocarcinoma HCA-7 cultures untreated (**A**) or treated with derivative **4** (**B**).

Preliminary fluorescence microscopy experiments were carried out *in vitro*. Since compounds **4** and **10** displayed the best COX-2 affinity and a suitable fluorescence, they were tested on some cancer cell lines. The results obtained in human epithelial colorectal adenocarcinoma HCA-7 cultures were promising (Figure 11). However, further studies on new cancer cell lines are needed to verify the possibility to use the derivative **4** or **10** as biomarkers to detect cancer or its progression.

## 3. Experimental

### 3.1. General Information

All commercial solvents and chemicals purchased from Aldrich, Fluka, Merck, Lancaster and Carlo-Erba were of analytic grade and used without further purification. NMR spectra (^1^H-NMR were recorded at 500 MHz, ^13^C-NMR were recorded at 125 MHz) on a Varian Instruments; chemical shifts (δ) are reported in ppm from tetramethylsilane as an internal standard; coupling constants (J) are in Hertz (Hz). IR spectra were recorded on a Perkin Elmer 1600 Series FT-IR in potassium bromide disks. Microanalyses for C, H, N and S were obtained from an EA 1108 elemental analyzer Fisons Carlo-Erba instrument. Analyses indicated by the symbols of the elements were within ±0.4% of the theoretical values. Melting points were determined in open capillary tubes on a SMP1 apparatus (Stuart Scientific Staffordshire) and are uncorrected. The purity of substances was checked by thin layer chromatography on Merck silica gel 60 F-254 plates. Mass spectra were recorded by Perkin Elmer Turbo Mass Clarus 560 Mass Spectrometer, with a 70 eV working ionization energy, source temperature 250 °C, trap current 90 μA. Synthesis and characterization of investigated compounds were reported previously [2].

### 3.2. Computational Method

It is known that the structure of human COX-2 is very similar to the murine enzyme [24]. To perform the protein-ligand modelling, the X-ray crystallographic 3D structure of murine COX-2 (mCOX-2) complex with naproxen was downloaded from the online Protein Data Bank (PDB ID: 3q7d) [28]. The inhibitor was subsequently removed and the structure subjected to energy minimization. The ligands were then built as pdb files by means of the server PRODRG2 [28].

The energies of the protein and the inhibitors were minimized by steepest descent algorithm. Simulations were performed by using the GROMACS simulation package (4.5.5) and GROMOS force-field parameter set 53A6. Force field was used to describe atomistically receptor, ligands, and ions, respectively [29,30].

Prior to dock inhibitors into target, naproxen was used as an inhibitor to mCOX-2 (PDB: 3q7d) to ensure whether the method was valid. The amino acid residues that bind compounds produced by the re-docking process were then compared with amino acid residues that bind crystal molecules; a root-mean-square deviation (RMSD) that was less than or equal to 2.0 Å was defined as reasonable criteria [31]. The value of RMSD for mCOX-2 obtained by us was 0.910 Å. This indicated that the parameter set for docking was suitable of reproducing the X-ray structure. In addition, the re-docking result for naproxen demonstrated that ligand invades COX-2 receptor-binding pockets, and that hydrogen bonds occur between inhibitors and carboxylic group of amino acid residue of Arg-120 and Tyr-355. These results were consistent with those reported before [24,32,33].

All docking calculations were carried out using software AutoDock 4.0. It is one of the most suitable methods for performing molecular docking of ligands to their macromolecular receptors, as well as to discriminate potential inhibitors. The ligand molecule is in an arbitrary conformation, orientation, and position and this molecular docking program [34] finds favourable poses in a protein-binding site using Lamarckian genetic algorithms implemented therein to search for the best conformers. The free energy of binding (ΔG) of mCOX-2 (prostaglandin synthase-2) complex with the inhibitors **1**–**11** was generated using this molecular docking program.

For mCOX-2 preparation, polar hydrogen were added, and then Kollman United Atom charges and atomic solvation parameters assigned. For ligands preparation, Gasteiger partial charges were added, non-polar hydrogen atoms merged, and rotatable bonds defined. The grid maps of docking studies were computed using the Autogrid program [31]. The grid must surround the region of interest in the macromolecule. The spacing between grid points was 1 Å for blind docking and 0.37 Å for active site, respectively. All parameters used in docking were default. A preliminary blind docking study was performed in order to discriminate the preferential binding sites of the ligand to the receptor. For the first simulation, in fact, the grid size was properly set up in order to contain the entire receptor structure up (40, 40, 40 Å along x, y and z, respectively, space between grid points of 1 Å).

For successive simulation, the pose of the ligand was selected in the active site and the grid was centered on this catalytic active region of the receptor (50, 50, 50 Å along x, y and z, respectively, space between grid points of 0.37 Å).

### 3.3. Absorption and Emission Spectroscopy

Absorption spectra were recorded with a HP (8452A) UV-VIS spectrophotometer, and fluorescence spectra were obtained with a Spex Fluorolog-2 (Mod F-111) spectrofluorometer. Molar extinction coefficients (ε) were determined for each final compound dissolved in DMSO, with concentration ranging from 1 to 50 μM and absorbance spectra recorded from 200 to 800 nm in standard quartz cuvettes. Epsilon values were determined by fitting the Beer’s law: A = ε c d where (A) is the absorbance at the λ_exc_; (c) is the molar concentration of the solution, and (d) was the optical path length (d = 1 cm). Measurements were repeated three times.

Emission spectra of derivatives **1**–**11** were determined in DMSO solution. In all experiments, the excitation and the emission bandpass was set at 2.5 nm and at 5 nm, respectively. The emission spectra were obtained from 388 to 700 nm, with excitation wavelength set at 368 nm. The excitation spectra of the compounds were obtained from about 250 to 450 nm, with the emission being recorded at the appropriate wavelength. Fluorescence quantum yields were calculated with respect to quinine sulphate (Fluka) in 0.5 M H_2_SO_4_ as a standard (Φ = 0.546) [35]. Solutions of both the sample and the reference were prepared from original solutions diluted with the appropriate solvent so that absorbance was below 0.1 at the same excitation wavelength (368 nm). Fluorescence measurements were carried out for each solution with the same instrument parameters, and the fluorescence spectra were corrected for instrumental response before integration. The quantum yield Φ_x_ for each sample was calculated according the following equation [36]:

Φ_x_ = Φ_S_ (A_S_/A_x_) (F_X_/F_S_) (n_x_/n_s_)^2^

where Φ is the emission quantum yield, A is the absorbance at the excitation wavelength, F is the area under the corrected emission curve, n is the refractive index of the solvent for the sample (X) and the standard (S).

### 3.4. Fluorescence Microscopy

In order to identify intracellular fluorescence of compounds **4** and **10**, fluorescent procedures were carried out using human epithelial colorectal adenocarcinoma HCA-7 cultures. HCA-7 cell line was provided by American Type culture Collection (Manassas, VA, USA) and routinely maintained in Dulbecco’s Modified Eagle’s Medium (DMEM, Sigma-Aldrich, Milan, Italy) supplemented with 10% fetal calf serum, 1% glutamine, 100 U/mL penicillin, and 100 μg/mL streptomycin, and incubated at 37 °C in a humidified, 95% air/5% CO_2_ atmosphere. The medium was changed every 2–3 days. For experiments, 24 h before the cells were trypsinized (Trypsin-EDTA; Sigma-Aldrich), counted in a haemocytometer, and plated (2 × 10^5^/well) in a 12-well culture plate for 24 h. Hence, the medium was removed and replaced with medium without phenol red containing 100 μM of experimental compounds for 12 h. Cells were first washed with PBS and then fixed with 4% paraformaldheyde in PBS for 30 min. Digital images were acquired using a fluorescence microscope (OPTIKA, M.A.D. Apparecchiature Scientifiche, Milan, Italy) at λ_exc_ = 325–375 nm and λ_em_ = 440 nm.

## 4. Conclusions

Our study demonstrated that some derivatives of benzothieno[3,2-d] pyrimidine could be developed as a novel class of anti-inflammatory agents. They are synthesized by a simple, efficient and ecologically friendly method, exhibit interesting anti-inflammatory properties related to interactions with active sites of COX-2, and show good fluorescence characteristics. Thus, this new class of anti-inflammatory may be promising for fluorescence imaging of cancer cells that express the COX-2 isozyme.
